# Extracellular Vesicles and Plant-Derived Vesicles in Cancer: From Mechanisms to Clinical Translation

**DOI:** 10.3390/pharmaceutics18091098

**Published:** 2026-08-31

**Authors:** Shery Jacob, Namitha Raichel Varkey, Sai H. S. Boddu, Jigar N. Shah, Ibrahim Mustafa Abdi, Rekha Rao

**Affiliations:** 1 Department of Pharmaceutical Sciences, College of Pharmacy, Gulf Medical University, Ajman P.O. Box 4184, United Arab Emirates; 2025mdd01@mygmu.ac.ae (N.R.V.); 2024mdd04@mygmu.ac.ae (I.M.A.); 2 Department of Pharmaceutical Sciences, College of Pharmacy and Health Sciences, Ajman University, Ajman P.O. Box 346, United Arab Emirates; s.boddu@ajman.ac.ae; 3 Center of Medical and Bio-Allied Health Sciences Research, Ajman University, Ajman P.O. Box 346, United Arab Emirates; 4 Department of Pharmaceutics, Institute of Pharmacy, Nirma University, Ahmedabad 382481, India; jigar.shah@nirmauni.ac.in; 5 Department of Pharmaceutical Sciences, Guru Jambheshwar University of Science and Technology, Hisar 125001, India; rekhaline@gjust.org

**Keywords:** extracellular vesicles, exosomes, microvesicles, apoptotic bodies, cancer therapy, plant-derived extracellular vesicles

## Abstract

Extracellular vesicles (EVs) are naturally occurring nanoscale carriers that have gained attention as next-generation platforms for diagnostics, site-specific drug delivery, and tissue engineering owing to their high biocompatibility, minimal immunogenicity, and capacity to transport diverse bioactive cargo across biological barriers. This review discusses the classification, biogenesis, molecular constituents, and therapeutic properties of the major EV subtypes such as exosomes, microvesicles, and apoptotic bodies. It also highlights recent advances in EV engineering for cancer treatment, emphasizing immune modulation and targeted therapeutic delivery. Particular attention is given to plant-derived EVs, which have shown promise as scalable, low-toxicity nanotherapeutics with inherent bioactivity and effective drug delivery potential. Selected preclinical studies, recent patents, and ongoing clinical trials are also summarized, providing an up-to-date perspective on the clinical translation of EV-based technologies. Current challenges in EV isolation, characterization, scalable manufacturing, cargo loading, standardization, and regulatory approval, along with future directions for clinical translation, are summarized. Collectively, this review summarizes the growing applicability of EVs as next-generation platforms for precision medicine, targeted drug delivery, and regenerative therapies while identifying the major obstacles that must be addressed to facilitate their successful clinical translation.

## 1. Introduction

Extracellular vesicles (EVs) are naturally occurring, lipid bilayer-enclosed particles released by cells into the extracellular environment that are incapable of replication, as they do not contain a functional nucleus. They play a crucial role in intercellular communication by transporting a variety of bioactive molecules, including proteins, lipids, DNA, mRNA, and microRNAs, between cells [1,2]. Due to their natural biocompatibility, low immunogenicity, and ability to cross biological barriers, EVs have gained significant attention as potential biomarkers for disease diagnosis and as promising natural nanocarriers for drug delivery and regenerative medicine applications [3,4]. Recent advances have centered on engineering EVs to enable the site-specific delivery of diverse therapeutic cargo, including small molecules, proteins, lipids, metabolites, and nucleic acids (e.g., mRNAs, miRNAs, and siRNAs), thereby expanding their applications in precision medicine, targeted drug delivery, and tissue engineering [5,6,7]. This narrative review was conducted using a structured literature search to ensure comprehensive and transparent coverage of the topic. Appropriate studies were extracted from databases including PubMed, Scopus, Web of Science, and Google Scholar, mainly covering literature from 2010 to May 2026, with earlier breakthrough articles included where applicable. Search terms included combinations of “extracellular vesicles,” “exosomes,” “microvesicles”, apoptotic bodies, “drug delivery,” “cancer,” “patents’’, “clinical trials,” and “clinical translation.” Original research articles, reviews, meta-analyses, clinical studies, and recent patents published in English were included based on scientific relevance, methodological quality, and novelty. Conference abstracts, editorials, duplicate publications, non-English articles, and studies lacking sufficient experimental or clinical information were avoided. Additional applicable references were recognized through manual screening of the reference lists of qualified articles. Illustrative studies were selected based on their relevance to the scope of the review, representation of distinct EV-based strategies in cancer, methodological significance, and ability to clearly demonstrate key advances in EV engineering and therapeutic applications.

### Classification

EVs are typically classified into exosomes (30–150 nm), microvesicles (50–1000 nm), and apoptotic bodies (50–5000 nm) based on their particle size and biogenesis [8,9]. A comparative overview of the principal EVs is presented in Table 1, highlighting their distinguishing features, surface markers, and therapeutic potential. Apoptotic bodies (ApoBDs) are EVs produced during programmed cell death (apoptosis), distinguishing them from exosomes and microvesicles (MVs) that are released by viable cells [4,8]. Although ApoBDs have attracted increasing research interest, their biogenesis, cargo sorting mechanisms, and biological functions remain incompletely characterized. As a result, current EV-based therapeutic strategies predominantly focus on exosomes and MVs, which have been extensively studied and demonstrate significant potential as drug delivery systems and regenerative medicine platforms [1,8]. These vesicles regulate numerous physiological and pathological processes, such as immune responses, tissue repair, cell signaling, and disease progression [10]. Recent advances in EV research have led to the identification of exomeres, a distinct and abundant population of non-membranous nanoparticles with a diameter of approximately 35 nm [11]. Exosomes are nanosized EVs secreted by both healthy and malignant cells through the endosomal trafficking pathway [12]. The biological properties of exosomes are essentially a result of the unique properties of their cells of origin [13]. For example, the molecular composition of exosomes provides valuable insights into the physiological and pathological state of their parent cells, making them promising biomarkers for identifying cellular origin and monitoring disease progression. Exosomes are enclosed by a lipid bilayer and carry a diverse molecular cargo and other bioactive molecules inherited from their parent cells. Through their transfer of molecular constituents to recipient cells, exosomes facilitate intercellular communication and modulate a broad range of physiological and pathological processes, including immune regulation, tissue repair, cancer progression, and cellular homeostasis [3,14]. To standardize EV research and improve reproducibility, the International Society for Extracellular Vesicles (ISEV) established the Minimal Information for Studies of Extracellular Vesicles (MISEV) guidelines, beginning with MISEV2014 and subsequently updated through MISEV2018 and MISEV2023 to reflect advances in the field and promote methodological rigor, transparency, and standardization [2,15,16]. Therefore, robust characterization and standardized reporting are essential for distinguishing exosomes from other EV subtypes and accurately attributing their biological functions. In accordance with MISEV2023, the terms ‘exosome’ and ‘microvesicle’ should be used only when their biogenesis or subtype identity is adequately supported; otherwise, operational terms such as small EVs (sEVs), medium/large EVs, or the broader term EVs are preferred. In this review, the EV subtype and cellular source are therefore specified whenever established in the original study, whereas ‘EVs’ is retained for heterogeneous or incompletely resolved vesicle populations; effects of conditioned medium or the whole secretome are distinguished from those of isolated EV preparations.

## 2. Biogenesis of Extracellular Vesicles

### 2.1. Biogenesis of Microvesicles

Microvesicles (MVs), also known as ectosomes or microparticles, are produced through the direct outward budding and fission of the plasma membrane. MV biogenesis is initiated by localized outward budding and blebbing of the plasma membrane, culminating in membrane scission and the shedding of MVs into the extracellular space. Cargo delivery to budding MVs is facilitated by ARF6-regulated recycling pathways, which transport proteins, lipids, and nucleic acids to sites of vesicle formation [17,18]. The formation and release of MVs are governed by coordinated changes in membrane lipid composition and cytoskeletal remodeling. The redistribution of membrane phospholipids, particularly.

**Figure 1:**
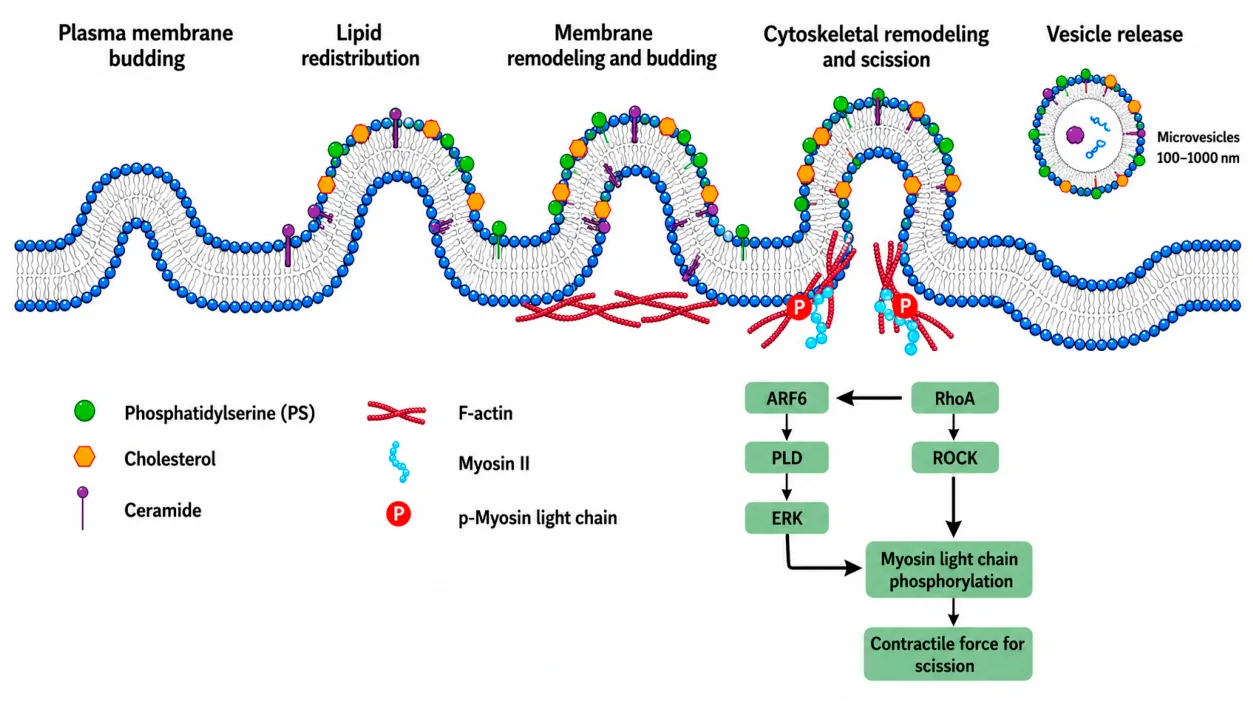
Biogenesis of microvesicles (MVs) by plasma membrane budding through lipid redistribution, membrane remodeling, and cytoskeletal contraction. Activation of the ARF6/PLD/ERK and RhoA/ROCK signaling pathways promotes myosin light chain phosphorylation, membrane scission, and MV release.

Phosphatidylserine externalization, in conjunction with cholesterol and ceramide enrichment, promotes membrane curvature, outward budding, and subsequent MV formation. MV biogenesis involves cytoskeletal remodeling and actomyosin-mediated contraction during vesicle scission. This process is regulated by the small GTPases ARF6 and RhoA, with ARF6 activating phospholipase D and ERK signaling, while RhoA stimulates the Rho-associated kinase (ROCK) signaling pathway. Activation of these pathways induces myosin light-chain phosphorylation, generating the contractile force required for microvesicle release [19,20]. MV formation is regulated by multiple factors, including calcium signaling, hypoxia, and endosomal sorting complex required for transport (ESCRT)-related proteins. Elevated calcium promotes membrane blebbing, hypoxia enhances Rab22A-mediated MV release, and ARRDC1, TSG101, and VPS4 contribute to the generation of distinct MV subpopulations [18,20]. Figure 1 provides a clear mechanistic overview of MV biogenesis, a fundamental process relevant to cancer biology and EV-based therapeutic approaches. It depicts MV formation through plasma membrane budding and highlights the coordinated roles of lipid redistribution, membrane and cytoskeletal remodeling, and the ARF6/PLD/ERK- and RhoA/ROCK-mediated signaling pathways involved in membrane scission and MV release. Understanding these molecular and cellular mechanisms provides an essential biological foundation for the subsequent discussion of the roles of EVs in cancer progression, intercellular communication, and therapeutic applications.

### 2.2. Biogenesis of Exosomes

**Figure 2:**
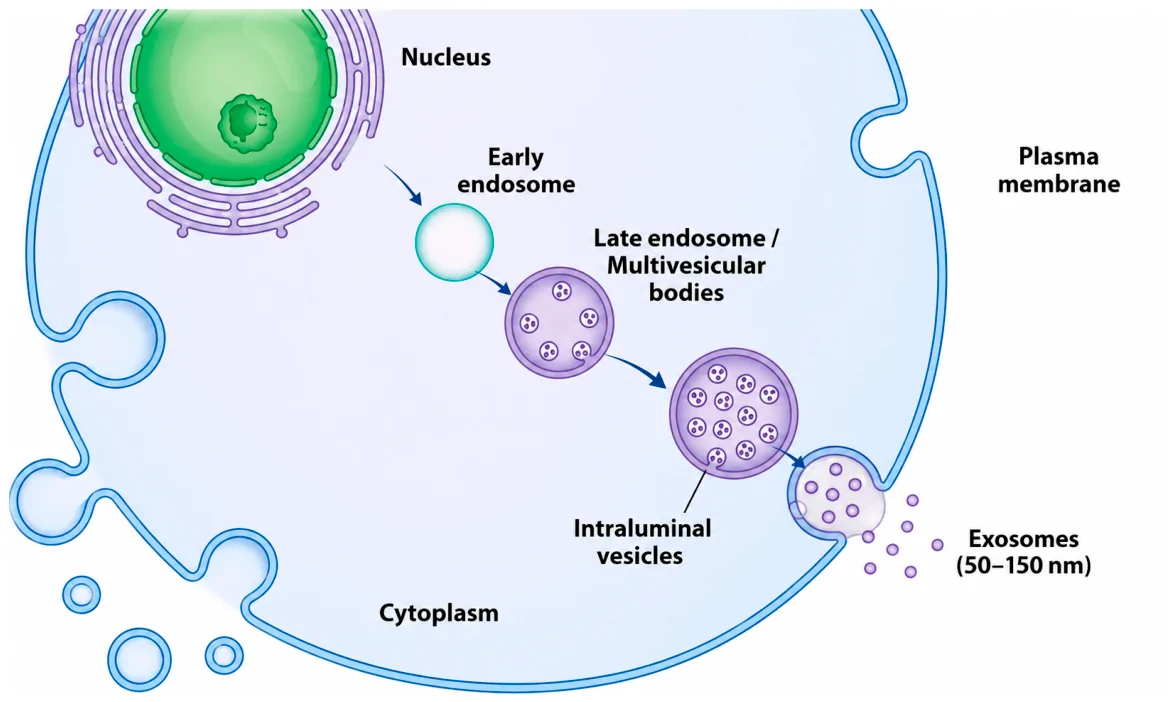
Biogenesis of exosomes through the endosomal pathway, where early endosomes mature into multivesicular bodies (MVBs) containing intraluminal vesicles (ILVs). Fusion of MVBs with the plasma membrane releases ILVs as exosomes into the extracellular space.

Exosome biogenesis is initiated by the inward budding of the endosomal membrane, resulting in the formation of intraluminal vesicles (ILVs) within multivesicular bodies (MVBs). Upon fusion of MVBs with the plasma membrane, the ILVs are released into the extracellular milieu as exosomes [21]. The ESCRT is essential for exosome biogenesis, mediating MVB formation, inward budding of ILVs and the selective sorting of cargo into ILVs [3,12]. The ESCRT machinery comprises four sequentially acting protein complexes (ESCRT-0, -I, -II, and -III). ESCRT-0 recognizes ubiquitinated cargo at the endosomal membrane and recruits ESCRT-I and ESCRT-II to promote membrane deformation and inward budding. ESCRT-III subsequently mediates membrane scission to form ILVs, after which it is disassembled by the ATPase Vps4 [22,23]. Several ESCRT-associated proteins, including Apoptosis-linked gene 2-interacting protein X (ALIX), tumor susceptibility gene 101 (TSG101), and charged multivesicular body protein 4 (CHMP4), have been identified in exosomes and contribute to membrane budding, cargo selection, and vesicle formation [24,25]. Besides the ESCRT-dependent pathway, exosome biogenesis can be mediated by ESCRT-independent mechanisms involving lipids and tetraspanins [26,27]. Tetraspanin-enriched microdomains (TEMs) facilitate cargo organization and protein loading into exosomes [28]. Actin cytoskeletal remodeling regulated by small GTPases, such as ADP-ribosylation factor 6 (ARF6), cell division control protein 42 homolog (Cdc42), and Ras-related proteins in the brain (Rab family of small GTPases), further supports inward vesicle budding within MVBs [29]. Although the final exocytosis of exosomes remains incompletely understood, Rab family GTPases (Rab11, Rab27, and Rab35) and SNARE proteins are recognized as key regulators of MVB fusion with the plasma membrane and exosome release [30,31]. Figure 2 illustrates the endosomal pathway of exosome biogenesis, providing a mechanistic basis for understanding the biological roles and therapeutic potential of EVs in cancer. It illustrates the maturation of early endosomes into MVBs, the formation and accumulation of ILVs within MVBs, and the subsequent fusion of MVBs with the plasma membrane, resulting in the release of ILVs as exosomes into the extracellular space. Understanding this biogenesis pathway provides an important biological foundation for the subsequent discussion of exosome-mediated intercellular communication, cancer progression, and their potential applications in cancer diagnosis and therapy.

### 2.3. Biogenesis of Apoptotic Bodies

**Figure 3:**
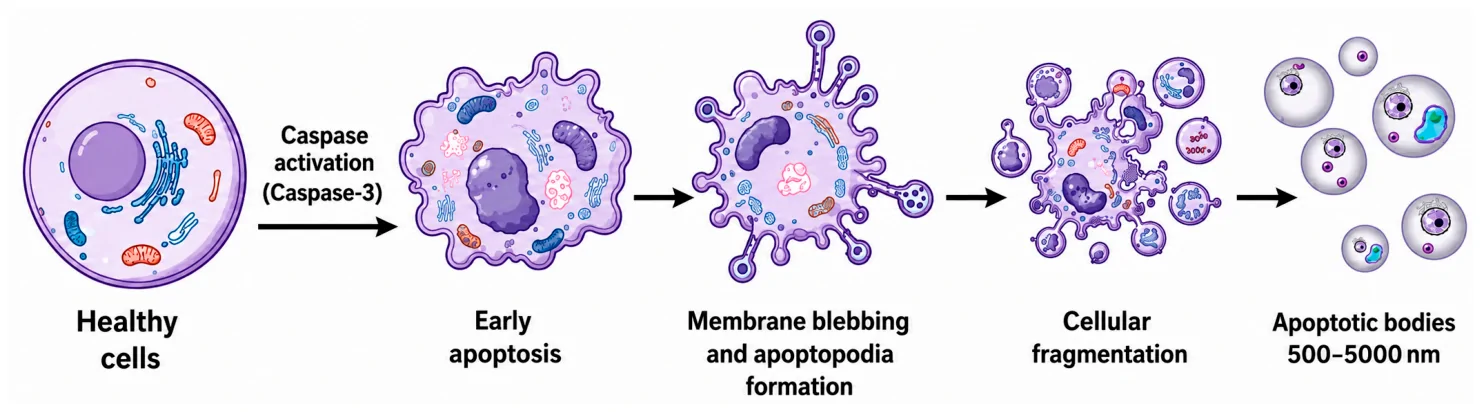
Biogenesis of apoptotic bodies during apoptosis, in which caspase-3 activation triggers membrane blebbing, apoptopodia formation, cellular fragmentation, and the release of membrane-bound apoptotic bodies.

Apoptotic bodies (ApoBDs) are EVs occurred during the late stages of programmed cell death (apoptosis). They represent the largest subclassification of EVs, with a particle size ranging from 500 nm to 5 μm [19,32]. Their biogenesis is initiated by the activation of caspases, particularly caspase-3, which orchestrates the dismantling of cellular structures through proteolytic cleavage of cytoskeletal and nuclear proteins [33,34]. This process is accompanied by apoptotic volume decrease (AVD), chromatin condensation, nuclear fragmentation (karyorrhexis), and extensive plasma membrane blebbing. Actomyosin contraction mediated by ROCK and myosin light-chain phosphorylation drives membrane protrusion and bleb formation, while specialized membrane extensions known as apoptopodia facilitate cellular fragmentation into discrete vesicles [32]. During this process, cellular contents, including cytoplasmic components, DNA fragments, organelles, and lipids, are packaged into ApoBDs [33]. Phosphatidylserine (PS) externalization on the outer plasma membrane is a hallmark of ApoBDs, promoting their recognition and clearance by phagocytes [19]. Following their release, ApoBDs are rapidly engulfed by macrophages and other phagocytic cells through efferocytosis, thereby preventing the release of intracellular contents and maintaining tissue homeostasis [33]. In addition to their role in cellular clearance, emerging evidence suggests that ApoBDs participate in intercellular communication via the transport of biomolecular cargos, thereby influencing immune regulation, tissue repair, and disease progression [32]. Figure 3 provides a mechanistic overview of the biogenesis of ApoBDs during apoptosis, highlighting a distinct pathway of extracellular vesicle formation relevant to cancer biology. It illustrates how caspase-3 activation induces membrane blebbing, apoptopodia formation, and cellular fragmentation, ultimately resulting in the release of membrane-bound apoptotic bodies containing cellular constituents. Understanding this process provides an important biological foundation for elucidating the roles of ApoBDs in the transfer of cellular components, intercellular communication, modulation of the tumor microenvironment, and their emerging diagnostic and therapeutic relevance in cancer.

## 3. Extracellular Vesicles in Cancer

Tumor-derived EVs (TDEVs) contribute to tumor progression by remodeling the tumor microenvironment and promoting tumor cell proliferation, angiogenesis, immune evasion, invasion, metastasis, and therapeutic resistance. TDEVs, which may comprise heterogeneous EV subtypes unless specifically resolved, transfer oncogenic cargo, including proteins, peptides, lipids, DNA, and diverse RNA species, from donor to recipient cells [35]. This molecular exchange can reprogram stromal cells, fibroblasts, endothelial cells, and immune cells, ultimately generating a tumor-supportive microenvironment that promotes cancer growth, progression, invasion, and metastasis [36]. Beyond their role in tumor progression, EVs have emerged as promising biomarkers for cancer detection and prognosis, as their molecular cargo reflects the molecular characteristics of the parent tumor cells. Engineered EVs are increasingly being explored as site-specific delivery platforms for the selective transport of chemotherapeutics, nucleic acids, and immunotherapeutic agents, offering enhanced targeting accuracy and reduced systemic toxicity for precision oncology.

EV-mediated communication in cancer is bidirectional: while tumor and tumor-microenvironment cells release outgoing pro-tumorigenic EVs, cancer cells also receive incoming EVs from stromal, immune, stem-cell, and other non-tumor sources. Incoming EVs can either support malignancy, as illustrated by adipocyte-derived exosomes that enhance fatty acid oxidation (FAO) and tumor-cell migration [37], or oppose it. CD8+ T cell-derived sEVs suppress tumor invasion and metastasis [38], IL2-tethered T cell-derived sEVs enhance antitumor CD8+ T-cell activity [39], human liver stem cell-derived EVs inhibit tumor angiogenesis [40], and adipose-derived stem cell EVs reduce glioblastoma proliferation, invasiveness, and angiogenic potential [41]. Thus, the biological outcome of EV signaling is direction-, source-, cargo-, and recipient-cell-dependent rather than uniformly pro-tumorigenic.

### 3.1. Extracellular Vesicle-Mediated Metabolic Reprogramming and Stromal Cell Remodeling

**Figure 4:**
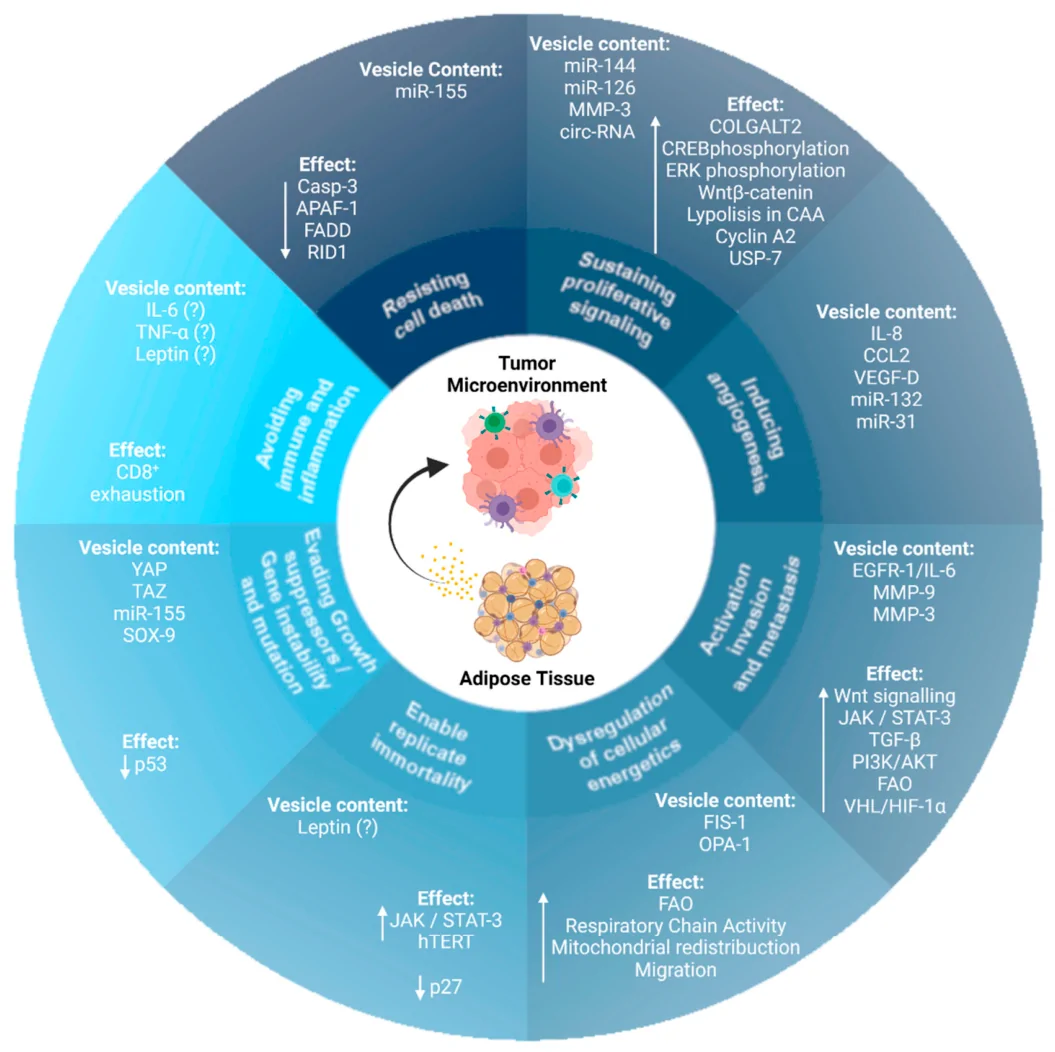
Schematic illustration of the role of adipose tissue-derived extracellular vesicles (EVs) in modulating the acquisition and maintenance of cancer hallmarks. Reproduced from Ref. [50].

Tumor-associated macrophages (TAMs) promote breast cancer glycolysis and chemoresistance by transferring HIF-1α-stabilizing long non-coding RNA (HISLA) via EVs, without specifying an EV subtype [42]. It was suggested that blocking EV-transmitted HISLA reduces glycolysis and chemoresistance, highlighting its therapeutic potential in breast cancer. EVs reprogram fibroblasts into cancer-associated fibroblasts (CAFs), promoting tumor progression through metabolic and signaling remodeling. Cancer-cell-secreted exosomal miR-105 metabolically reprograms CAFs, enabling them to support tumour growth by supplying nutrients in nutrient-rich conditions and recycling metabolic waste into energy-rich metabolites under nutrient-deprived conditions [43]. In nasopharyngeal carcinoma (NPC), EV-derived latent membrane protein 1 (LMP1) induces the conversion of fibroblasts into CAFs through nuclear factor kappa B (NF-κB) p65 (RelA) subunit activation, thereby promoting glycolysis, autophagy, tumor progression, metastasis, radioresistance, and the reverse Warburg effect [44]. Similarly, in oral cavity squamous cell carcinoma (OCSCC), tumour-derived MVs induce CAF formation via ERK1/2-mediated caveolin-1 degradation. This promotes aerobic glycolysis and lactate production, thereby enhancing cancer cell migration and invasion [45]. Kaposi’s sarcoma-associated herpesvirus (KSHV)-infected cells transfer viral RNAs via exosomes that stabilize HIF-1α in neighbouring cells, enhancing aerobic glycolysis and the production of energy-rich metabolites that support tumour growth [46]. Similarly, exosomes derived from mutant Kirsten rat sarcoma (KRAS)-mutant colorectal cancer cells transfer glucose transporter 1 (GLUT1), inducing a Warburg-like metabolic phenotype and enhancing the proliferation of recipient epithelial cells [47]. Tumor-microenvironment-derived exosomes can reprogram cancer cell metabolism by supplying nutrients and altering oxidative phosphorylation [48]. Tumour-associated macrophage-derived EVs enhance glycolysis and lactate production, establishing positive feedback loops that sustain tumor growth [49]. TDEVs regulate macrophage polarization, altering the M1/M2 balance and consequently influencing tumor progression. Adipose tissue-derived EVs play a key role in obesity-associated cancer by mediating communication between adipocytes and tumor cells [50,51]. Through local and systemic EV-mediated signaling, adipose tissue contributes significantly to the tumor microenvironment and enhances cancer malignancy. A schematic overview of the role of adipose tissue-derived EVs in modulating the acquisition and maintenance of key cancer hallmarks is presented in Figure 4. The figure illustrates the multifaceted involvement of these EVs in tumor-associated processes, including cancer cell proliferation and survival, angiogenesis, invasion and metastasis, metabolic reprogramming, immune modulation, and alterations in the tumor microenvironment. In particular, adipocyte-derived EVs transfer FAO-related proteins to cancer cells, thereby enhancing FAO and increasing mitochondrial abundance without significantly affecting glycolysis. By integrating these EV-mediated molecular and metabolic mechanisms into a comprehensive framework, Figure 4 facilitates an understanding of the contribution of adipose tissue-derived EVs to cancer development and progression while highlighting their potential relevance as diagnostic biomarkers and therapeutic targets. In this context, adipocyte-derived exosomes promote fatty-acid oxidation and enhance melanoma cell migration and invasion [37], further supporting their role in tumor progression. Collectively, these results demonstrate that EV-mediated metabolic reprogramming and stromal cell remodeling are central drivers of tumor progression, highlighting EV-associated signaling pathways as promising therapeutic targets for disrupting the tumor-supportive microenvironment. Taken together, these studies demonstrate that although EV cargo and signaling pathways vary across cancer types, they converge on common biological outcomes, including metabolic reprogramming, stromal remodeling, immune modulation, and enhanced therapeutic resistance. This comparative evidence suggests that targeting shared EV-mediated metabolic regulators, such as HIF-1α signaling, glycolytic pathways, CAF activation, or EV biogenesis and uptake, may offer broader therapeutic benefits than approaches directed at individual tumor-specific molecules.

### 3.2. Extracellular Vesicles in Immune Modulation and Tumour Immune Evasion

EVs play a central role in tumor immune modulation by facilitating communication between cancer and immune cells, thereby promoting immune evasion, suppressing antitumor responses, and contributing to resistance against immunotherapy. Cancer cell-derived small EVs (CA-sEVs) express programmed death-ligand 1 (PD-L1) similarly to their parent cells. Notably, PD-L1-bearing EVs secreted by metastatic melanoma cells suppress cluster of differentiation 8-positive (CD8^+^) T-cell responses, facilitating immune escape and tumor expansion [52]. Exosomal PD-L1 (ePD-L1) promotes tumour immune evasion by suppressing T-cell activity and contributes to resistance against immune checkpoint therapy through interaction with PD-1 on CD8^+^ T cells, thereby reducing their antitumour effects [53,54]. TDEVs promote immune evasion by suppressing natural killer (NK) cell activity [55] and PD-L1 expression. They also increase lactate production, leading to extracellular acidosis that further impairs NK cell proliferation and function [56]. Therapeutic strategies aimed at reducing exosomal PD-L1 (ePD-L1) expression or inhibiting sEVs release have been suggested to enhance the efficacy of anti-PD-1/PD-L1 immunotherapy by overcoming treatment resistance. Immune cell-derived small extracellular vesicles (IM-sEVs) play a pivotal role in immune regulation by mediating crosstalk between innate and adaptive immune cells, highlighting their potential as novel agents for cancer immunotherapy [57,58,59]. For instance, CD8^+^ T cell-derived sEVs can suppress tumour invasion and metastasis by depleting mesenchymal stem cells within the tumour microenvironment [38], whereas CD4^+^ T cell-derived sEVs promote B-cell activation and strengthen antigen-specific humoral immune responses [60]. Jurkat T cell-derived interleukin-2-tethered small extracellular vesicles (IL2-sEVs) enhanced CD8^+^ T-cell antitumor activity and reduced PD-L1 expression in melanoma cells, thereby sensitizing them to T cell-mediated cytotoxicity [39]. These effects were mediated by interleukin-2 (IL-2)-induced miRNAs, particularly miR-181a-3p and miR-223-3p, which decreased PD-L1 concentration, while miR-181a-3p additionally promoted CD8^+^ T-cell activity and inhibited regulatory T-cell function. IL2-sEVs effectively suppressed tumour growth in immunocompetent mice and showed enhanced therapeutic efficacy when combined with existing anticancer drugs, highlighting their potential as a novel cancer immunotherapy that modulates both immune and tumour cells through miRNA reprogramming. EVs contribute to T-cell immunosuppression by promoting adenosine-mediated signaling. EV-associated ectonucleotidases, such as CD39 and CD73, catalyze the conversion of extracellular ATP into adenosine, which subsequently interacts with A1, A2A, A2B, and A3 adenosine receptors expressed on immune cells [61]. Activation of these receptors, particularly A2A and A2B, inhibits T-cell activation and effector functions, thereby facilitating immune suppression and tumor immune escape. TEVs containing arginase-1 (ARG1) enhance immune suppression in ovarian cancer by limiting the expansion of CD4^+^ and CD8^+^ T cells, thereby facilitating tumor immune escape [62]. Hypoxia-induced TDEVs stimulate M2 macrophage polarization and elevate cyclooxygenase-2 (COX-2), prostaglandin E synthase-1 (PGES-1), and interleukin-6 (IL-6) expression, thereby promoting immune suppression and tumor progression [63]. Engineered EVs from different types of cancers can repolarize M2 macrophages toward the antitumor M1 phenotype, thereby enhancing immune responses and offering a promising approach for cancer therapy [64]. These EVs are transported to draining lymph nodes, where they impair antigen-specific T-cell responses, driving tumor growth and immune escape. Colorectal cancer (CRC)-derived sEVs, particularly those carrying miR-21-5p and miR-200a, induce macrophage polarization toward an immunosuppressive PD-L1^+^CD206^+^ M2-like phenotype through activation of the PTEN/AKT (phosphatase and tensin homolog/protein kinase B) SOCS1/STAT1 (suppressor of cytokine signaling 1/signal transducer and activator of transcription 1) signaling pathways [65]. These polarized macrophages suppress CD8^+^ T-cell activity, fostering tumor progression, and are linked to poor prognosis. Targeting CRC-derived sEV-miRNAs or PD-L1^+^ TAMs may hold promise as a therapeutic strategy to enhance antitumor immunity and increase the effectiveness of anti-PD-L1 therapy. Thus, targeting EV-mediated immune signaling or exploiting engineered EVs as immunomodulatory nanocarriers represents a promising strategy for overcoming tumor immune escape and improving clinical responses to cancer immunotherapy.

In short, the existing data indicates that despite differences in EV origin and molecular cargo, TDEVs converge on common biological outcomes, including suppression of cytotoxic lymphocytes, polarization of macrophages toward immunosuppressive phenotypes, activation of immune checkpoint pathways, and metabolic inhibition of antitumour immunity. Conversely, immune cell-derived and engineered EVs exhibit predominantly immunostimulatory properties capable of reversing these effects. This functional contrast highlights EVs as both mediators of tumour immune escape and versatile therapeutic platforms, supporting the development of EV-targeted interventions and engineered EV-based immunotherapies to enhance the efficacy of current cancer treatments.

### 3.3. Extracellular Vesicles in Angiogenesis and Therapeutic Applications

TDEVs enhance tumor angiogenesis by delivering pro-angiogenic cargo to endothelial cells, thereby promoting tumor growth and metastasis [66]. Furthermore, TDEVs remodel the tumor microenvironment by delivering bioactive molecules, including proteins, lipids, and microRNAs, that activate endothelial cells and promote growth, translocation, and tube formation. Cancer-derived MVs deliver Hsp90-stabilized vascular endothelial growth factor (VEGF) 90K, which activates VEGF receptors and promotes neovascularization, thereby enhancing tumor growth and progression [67]. For example, pancreatic cancer (PC)-derived EVs carrying IL-17B activate pancreatic stellate cells (PSCs), leading to increased IL-17BR expression, enhanced oxidative phosphorylation (OXPHOS), and reduced mitochondrial turnover. Activated PSCs subsequently promote tumor cell metabolism through IL-6-mediated signaling, creating a reciprocal feedback loop that further enhances tumor growth [68]. Emerging evidence also suggests that T-EVs contribute to resistance against anti-angiogenic therapy (AAT), limiting its long-term clinical efficacy. Consequently, targeting EV-mediated signaling, particularly T-EVs, has been proposed. Consistent with the bidirectional nature of EV communication, incoming EVs from non-tumor sources can counteract tumor angiogenesis. Human liver stem cell-derived EVs (HLSC-EVs) suppress the angiogenic behavior of tumor endothelial cells by delivering specific miRNAs, including miR-15a, miR-181b, miR-320c, and miR-874 [40]. By targeting pro-angiogenic genes, including ITGB3, FGF1, EPHB4, and PLAU, these miRNAs reduce angiogenesis and impede tumour growth. Adipose tissue-derived mesenchymal stem cell EVs exhibit antitumor activity against glioblastoma by reducing tumor cell proliferation, invasiveness, and angiogenic potential [41]. Adipose-derived stem cell EVs (ASC-EVs) downregulate genes associated with cell adhesion, invasion, and vascularization, leading to decreased tumor growth and neovascularization. Therefore, targeting pro-angiogenic EV signaling or utilizing therapeutic EVs with anti-angiogenic properties may represent an effective strategy for inhibiting tumor vascularization and enhancing the efficacy of cancer therapies.

Overall, current experimental findings demonstrate a clear functional contrast between tumour-derived and therapeutic EVs. Whereas TDEVs promote angiogenesis, stromal remodeling, and therapeutic resistance, stem cell-derived EVs suppress vascularization and tumour progression through coordinated inhibition of multiple angiogenic pathways. These comparative findings support the development of dual therapeutic strategies that combine inhibition of pro-angiogenic TDEVs with the administration of engineered or stem cell-derived EVs to overcome resistance to anti-angiogenic therapy and improve clinical outcomes.

### 3.4. Role of Plant-Derived Vesicles in Cancer

**Figure 5:**
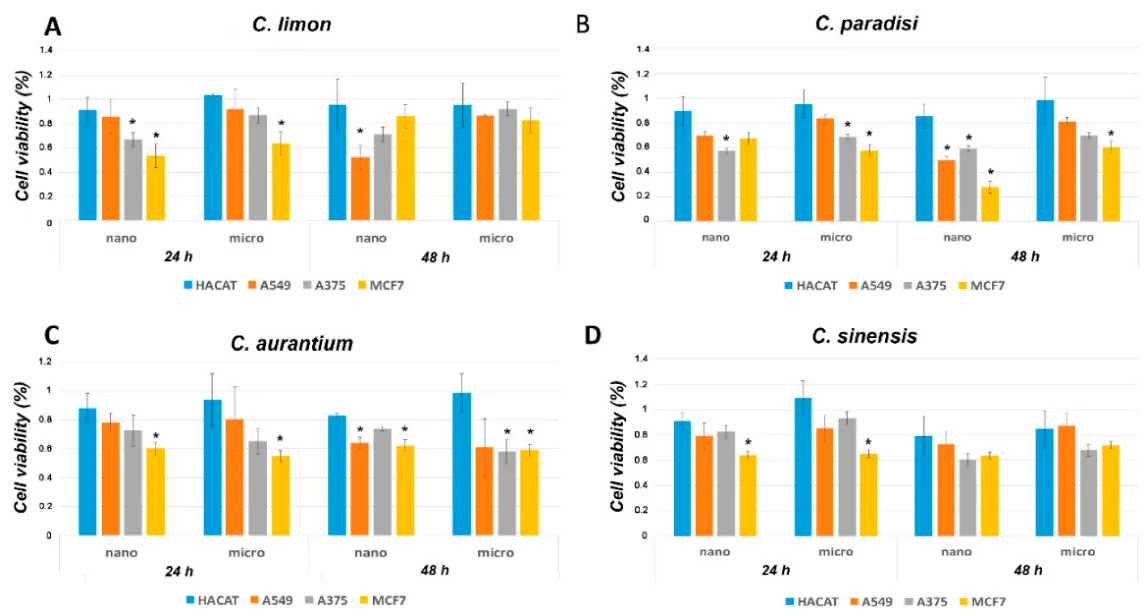
Nano- and microvesicles isolated from the fruit juices of four Citrus species inhibited the proliferation of cancer cells after 24 and 48 h of treatment at a concentration of 25 μg/mL. Cell viability was evaluated using the MTT assay and expressed relative to untreated control cells (100% viability). (**A**) *C. limo*n; (**B**) *C. paradisi*; (**C**) *C. aurantium*; (**D**) *C. sinensis*. Data are presented as the mean ± SD of three independent experiments. Asterisks (*) indicate statistically significant differences compared with non-tumour HaCaT cells (*p* ≤ 0.05; Student’s *t*-test). Reproduced from Ref. [72].

Plant-derived extracellular vesicles (PD-EVs) have emerged as promising natural nanotherapeutics for cancer treatment owing to their intrinsic anticancer activity, excellent biocompatibility, low toxicity, and ability to deliver endogenous or therapeutic cargo to tumor cells. PD-EVs can contribute to cancer therapy through two complementary approaches. First, native PD-EVs may function as therapeutic agents themselves, as their endogenous cargo including lipids, proteins, nucleic acids, and plant secondary metabolites can exert intrinsic biological and anticancer effects by modulating tumor cell proliferation, apoptosis, oxidative stress, immune responses, and the tumor microenvironment. Second, PD-EV-derived nanovectors can serve as carriers for therapeutic agents. For example, grapefruit-derived nanovectors have been engineered with activated leukocyte membranes for targeted delivery of therapeutic agents to inflammatory tumor sites [69]. This dual functionality as both intrinsically bioactive vesicles and biocompatible delivery vectors distinguishes PD-EVs as a versatile platform for cancer therapy [70]. PD-EVs, especially those harvested from *Dendropanax morbifera* (DM-EVs) and *Pinus densiflora*, demonstrated selective antineoplastic effects. Combination therapy with DM-EVs and PD-EVs produced a synergistic inhibitory activity against breast and skin cancer cells; in cancer cell lines (MDA-MB-231, MCF7, and A431), the combination index (CI) values were below 0.5, indicating strong synergism [71]. MVs and nanovesicles (NVs) extracted from four *Citrus* species (*C. sinensis*, *C. aurantium*, *C. limon*, and *C. paradisi*) were assessed for their effects on the proliferation of HaCaT, A375, A549, and MCF-7 cell lines [72]. Figure 5 shows that Citrus-derived MVs and NVs exhibited minimal toxicity toward normal HaCaT cells while selectively reducing the viability of various cancer cell lines, demonstrating promising anticancer potential. The figure provides comparative and statistically validated evidence of the anticancer effects of *Citrus*-derived vesicles relative to non-tumour HaCaT cells. These vesicles suppressed cancer cell proliferation by promoting G2/M cell cycle arrest via the downregulation of cyclins B1 and B2 and the upregulation of the cell cycle inhibitor p21. Their anticancer effects were further mediated by inhibition of the Akt and ERK signaling pathways, downregulation of intercellular adhesion molecule-1 (ICAM-1) and cathepsins, and enhanced PARP-1 cleavage, thereby promoting apoptosis and suppressing tumor progression.

Aloe vera-derived EV-like particles (AV-EVLPs) exhibited potent anti-pancreatic cancer activity by inhibiting Panc-1 cell proliferation, migration, and invasion while promoting reactive oxygen species (ROS) generation and pyroptosis-related inflammatory responses [73]. In a pancreatic cancer xenograft model, the unmodified EVs (EV-U) formulation significantly reduced tumor growth without detectable toxicity. Its antitumor effects were attributed to activation of the caspase-1/3/7/9-GSDMD/E-mediated pyroptosis pathway, suggesting AV-EVLPs as a promising and safe therapeutic approach for pancreatic carcinoma. Increasing experimental findings indicate that plant-derived extracellular vesicles (PD-EVs) act as natural nanotherapeutics by transporting endogenous bioactive molecules that confer intrinsic therapeutic effects across diverse disease models, eliminating the need for additional drug loading [74]. Ginger-derived exosome-like nanovesicles (GDENs) are recognized as a versatile, inexpensive substitute to mammalian exosomes for targeted siRNA delivery [75]. Folic acid-functionalized GDENs, engineered using arrowtail RNA nanoparticles, effectively delivered surviving siRNA to tumor cells and achieved gene silencing comparable to that of conventional transfection approaches. Intravenous administration in a xenograft model significantly inhibited tumor growth. Garlic-derived small EVs (SEVs) exhibited selective anticancer activity by inducing caspase-mediated apoptosis and reducing VEGF expression in kidney and lung cancer cells, while preserving normal cells [76]. Brucea javanica-derived exosome-like nanovesicles (BF-Exos) exhibited potent therapeutic activity against triple-negative breast cancer (TNBC), a highly aggressive subtype with limited treatment options [77]. BF-Exos transferred 10 functional microRNAs (miRNAs) to TNBC cells, inhibiting tumor growth and metastasis by downregulating the PI3K/Akt/mTOR signaling pathway and enhancing ROS/caspase-regulated apoptosis. They also reduced VEGF secretion, thereby inhibiting tumor angiogenesis. Taken together these investigations reveal that PD-EVs combine intrinsic anticancer activity with efficient therapeutic cargo delivery, highlighting their potential as safe, biocompatible, and multifunctional nanoplatforms for next-generation cancer therapy. Table 2 summarizes representative studies on PD-EVs for cancer therapy, highlighting their sources, experimental models, mechanisms of action, and therapeutic outcomes.

Taken together, comparative evaluation of current studies demonstrates that although PD-EVs differ in botanical source, molecular composition, and therapeutic strategy, they consistently exhibit excellent biocompatibility, selective tumour targeting, and multimodal anticancer activity. Their ability to integrate intrinsic therapeutic properties with efficient cargo delivery positions PD-EVs as versatile nanoplatforms for next-generation cancer therapy.

## 4. Patent

The expanding patent landscape highlights the growing translational and commercial potential of EV-based technologies across various cancer types. Representative patents related to cancer therapeutics are presented in Table 3. These inventions encompass several key areas, including enhancement of EV production, selective isolation and detection of tumor-derived EVs, cancer immunotherapy and vaccination, and the development of EVs as intrinsic therapeutic agents and drug-delivery platforms. One important area of innovation is the development of strategies for improving EV production and therapeutic cargo delivery. Patent 116113690 describes the use of a Noxa protein-derived peptide to promote efficient, high-yield EV production. The resulting EVs can facilitate the delivery of diverse therapeutic cargo, including proteins, plasmid DNA, and membrane-impermeable drugs, highlighting the potential of this approach to address production and cargo-loading requirements associated with the clinical translation of EV-based therapeutics. In parallel, several patents exploit the disease-specific molecular characteristics of tumor-derived EVs for diagnostic applications. For example, WO/2024/225847 employs antibody-based approaches targeting cancer-specific surface markers to selectively isolate breast cancer-derived EVs and enable subsequent microRNA extraction for diagnostic and research purposes. Patent activity also demonstrates considerable interest in harnessing EVs for cancer immunotherapy. Patent 1020220156460 describes immune cells, including chimeric antigen receptor (CAR)-expressing cells, engineered through MAL and/or CD9 expression to enhance EV secretion, thereby improving cancer immunotherapeutic efficacy. Another approach is represented by 112870340, which describes a breast cancer EV-based tumor vaccine incorporating Toll-like receptor stimulants and mannose-modified lipids. The formulation is designed to enhance antitumor immunity through immune-cell activation, thereby inhibiting tumor growth and supporting EV-based cancer immunotherapy. In addition to mammalian cell-derived EVs, recent patents demonstrate increasing exploration of biologically derived vesicles from microorganisms and edible plants as alternative anticancer platforms. Patent 119060911 reports EVs derived from Lactobacillus salivarius LZZAY01 that exhibit anti-colon cancer activity while promoting normal intestinal epithelial cell growth, supporting their potential application in cancer prevention and therapy. Likewise, 1020240116412 describes EVs derived from edible crops that possess intrinsic anticancer activity while simultaneously functioning as natural drug-delivery systems. Such characteristics broaden the potential utility of EVs by combining biological activity with carrier functionality for cancer prevention and treatment.

## 5. Clinical Translation

The expanding translational potential of EVs is evidenced by the increasing number of clinical trials investigating their applications across various cancer types. Table 4 summarizes the current clinical landscape of EV-based studies in oncology, including study type or phase, cancer indication, enrolment, and clinical trial identifiers. These studies demonstrate the versatility of EVs as diagnostic, prognostic, and predictive biomarkers, as well as their potential role in guiding personalized cancer therapy. Several clinical trials are investigating EVs for predicting treatment-related outcomes and therapeutic responses. The CHEMOVES study (NCT07558447) evaluates circulating EV levels, lipids, and microRNAs as predictive biomarkers for chemotherapy-induced peripheral neuropathy in patients receiving neurotoxic chemotherapy. In non-small cell lung cancer (NSCLC), EVs are being explored alongside patient-derived organoids and PD-L1 PET imaging as biomarkers for predicting responses to immune checkpoint inhibitors, including pembrolizumab and dostarlimab (NCT06405230). Furthermore, EV-derived DNA obtained from bronchoalveolar lavage fluid (BALF) has been investigated for identifying T790M-positive NSCLC and guiding olmutinib therapy (NCT03228277). EV-based approaches are also being investigated for cancer diagnosis, molecular profiling, and personalized therapy. In myeloproliferative neoplasms, EVs and cellular senescence are being studied to identify sex-specific prognostic biomarkers and personalized therapeutic targets (NCT06798805). In breast cancer, glycosylated EVs and their contents are being evaluated as potential biomarkers for early disease detection (NCT05417048). Additionally, a Phase II study in resectable EGFR-mutated NSCLC evaluates neoadjuvant lazertinib while exploring EV-based BALF liquid biopsy as a minimally invasive approach for detecting EGFR mutations (NCT05469022). Collectively, these clinical investigations highlight the growing role of EVs across the cancer-management continuum, encompassing early detection, molecular characterization, prediction of treatment response and toxicity, mutation detection, and personalized therapeutic selection. These developments support the potential translation of EV-based technologies into clinically relevant tools for precision oncology. Notably, however, most ongoing clinical trials primarily evaluate the intrinsic therapeutic properties of EVs, whereas comparatively few investigate their utility as carriers for exogenous therapeutic cargo. Despite encouraging outcomes from preclinical and early clinical studies, the widespread clinical adoption of EV-based therapeutics remains limited by challenges related to biological heterogeneity, scalable manufacturing, quality assurance, and regulatory compliance [84]. A major obstacle to the clinical translation of EV-based therapeutics is the scalable production of clinical-grade EVs while ensuring batch-to-batch consistency, product purity, biological activity, and reproducible cargo composition. Variability arising from donor cell sources, culture conditions, isolation procedures, and storage methods further contributes to product heterogeneity, underscoring the need for standardized Good Manufacturing Practice (GMP)-compliant manufacturing processes and validated quality-control assays [85,86]. Clinical-grade EV manufacturing must also comply with internationally recognized quality standards to support product safety and regulatory approval. These include ISO 13485 for quality management systems, ISO 14644 for cleanroom manufacturing, and, where appropriate, ISO 10993 for biological safety and biocompatibility evaluation [87]. Maintaining sterility and storage stability is another critical requirement for clinical-grade EV production. Because conventional terminal sterilization methods may damage EV membranes and impair biological function, manufacturing should rely on aseptic processing, supplemented by sterile filtration where appropriate, together with validated storage protocols and rigorous stability studies to ensure product integrity throughout its lifecycle [88]. Regulatory approval of EV-based therapeutics is further challenged by their inherent biological complexity and the lack of harmonized international regulatory frameworks governing their development, manufacturing, and clinical evaluation [89]. To achieve regulatory approval, EV-based therapeutics must undergo rigorous characterization encompassing product identity, purity, potency, mechanism of action, biodistribution, safety, and manufacturing consistency. Establishing harmonized analytical methods, validated potency assays, and robust chemistry, manufacturing, and control (CMC) frameworks will therefore be pivotal for regulatory acceptance and successful commercialization [90]. High manufacturing costs associated with large-scale cell culture, EV isolation, purification, characterization, and quality control continue to limit the commercial feasibility of EV-based therapeutics. However, advances in bioreactor technology, scalable purification, automated manufacturing, and closed-system production are expected to improve production efficiency and reduce costs. Continued international collaboration among academia, industry, regulatory agencies, and the International Society for Extracellular Vesicles (ISEV) will be essential for developing standardized manufacturing and regulatory guidelines that accelerate the clinical translation of EV-based therapeutics [91].

## 6. Future Perspectives and Challenges

EVs have emerged as promising therapeutic and diagnostic platforms owing to their natural biocompatibility, low immunogenicity, ability to cross biological barriers, and capacity to transport diverse bioactive cargo [92]. Advances in EV engineering, together with improvements in cargo loading, surface modification, and targeted delivery strategies, have broadened the therapeutic applications of EVs in cancer treatment [93]. Compared with mammalian EVs, PDEVs are more readily available, amenable to large-scale production, cost-effective to manufacture, and possess intrinsic therapeutic activity [94,95,96]. Although significant progress has been made, several barriers continue to limit the clinical translation of EV-based therapeutics. In particular, the absence of standardized protocols for EV isolation, purification, characterization, storage, and quality control remains a major source of variability, affecting reproducibility and cross-study comparisons. While the minimal information for studies of EVs (MISEV) guidelines have established an important framework for standardization, further harmonization is required to ensure consistency across laboratories and facilitate clinical implementation [97]. Large-scale production of clinical-grade EVs remains a significant challenge because current isolation and manufacturing methods are constrained by low yield, product heterogeneity, and high production costs [98]. Conventional two-dimensional (2D) cell culture systems often yield insufficient quantities of EVs for large-scale applications. To address this limitation, scalable production platforms, including three-dimensional (3D) cultures, microcarrier-based systems, and bioreactors, have been developed to enhance EV productivity. Furthermore, purification techniques such as tangential-flow filtration and size-exclusion chromatography are increasingly employed to improve EV yield, purity, batch-to-batch consistency, and preservation of biological activity [99].

Sterility assurance represents another key hurdle in the clinical translation of EV therapeutics, as conventional terminal sterilization methods, such as heat treatment and irradiation, can compromise EV membrane integrity and biological activity. Consequently, the manufacture of clinical-grade EVs should be performed under aseptic conditions in cleanroom facilities compliant with ISO 14644-1-2015 [100], ISO-14644-2-2015 [101], ISO 14644-3:2019 [102], ISO 14644-4:2022 [103], and ISO 14644-5:2025 [104], supported by quality management systems aligned with ISO 13485 [105] and, where applicable, biological safety evaluation according to ISO 10993 [106]. Optimizing storage conditions is equally important, as repeated freeze–thaw cycles, prolonged storage, and inappropriate formulation buffers can promote EV aggregation, compromise membrane integrity, degrade cargo molecules, and diminish therapeutic efficacy [107,108]. Standardized storage protocols, optimized cryoprotective formulations, and comprehensive stability studies are therefore essential to preserve EV quality throughout manufacturing, storage, and distribution. Equally important is the development of validated potency assays and well-defined critical quality attributes that can reliably predict therapeutic performance and ensure batch-to-batch consistency [109,110]. Further optimization of cargo loading, targeted delivery, and controlled release strategies is essential to maximize the therapeutic potential of EVs. Although engineered EVs have demonstrated enhanced therapeutic efficacy, key challenges—including limited loading efficiency, cargo instability, suboptimal biodistribution, variable pharmacokinetics, and uncertain long-term safety remain to be addressed [111]. The commercialization and clinical adoption of EV-based therapeutics are further impeded by the lack of well-defined regulatory frameworks governing their development, manufacturing, quality control, and clinical evaluation [112].

The inherent biological complexity and heterogeneity of EV populations also complicate the identification of the specific bioactive components responsible for their therapeutic effects. In addition, the lack of comprehensive regulatory frameworks governing EV manufacturing, quality control, safety assessment, and clinical evaluation continues to impede their commercialization and clinical approval [98]. Future efforts should prioritize the development of scalable manufacturing platforms, advanced EV engineering technologies, standardized quality-control frameworks, and well-designed clinical trials to establish long-term safety and therapeutic efficacy. Emerging innovations in synthetic biology, nanotechnology, artificial intelligence-assisted cargo engineering, and precision medicine are expected to further advance the design and clinical translation of next-generation EV therapeutics. Moreover, integrating EVs with biomaterials, hydrogels, and tissue-engineered scaffolds offers exciting opportunities to enhance targeted drug delivery, tissue regeneration, and personalized regenerative medicine [96]. Ultimately, the successful clinical translation and commercialization of EV-based therapeutics will depend on adequately powered, multicenter randomized clinical trials incorporating standardized dosing regimens, validated potency assays, and comprehensive long-term safety monitoring.

## 7. Conclusions

The growing understanding of EV biology has positioned these naturally occurring nanocarriers as promising platforms for disease diagnosis, precision drug delivery, and regenerative medicine [113,114]. EVs play a central role in intercellular communication by transporting diverse bioactive cargo, including proteins, lipids, and nucleic acids, thereby influencing numerous physiological and pathological processes [98]. Emerging evidence underscores the diverse biological functions of EVs in cancer, immune regulation, neuroprotection, tissue regeneration, and wound healing. Concurrent advances in EV engineering, phytochemical loading, and the development of plant-derived EVs have further expanded their therapeutic potential, establishing EVs as versatile and increasingly translatable nanotherapeutic platforms [94,96,115]. The expanding body of preclinical evidence, ongoing clinical trials, and increasing patent activity collectively demonstrate the advancing clinical translation of EV-based technologies [84]. The successful clinical implementation of EV-based therapeutics will depend on overcoming several key challenges, including the establishment of standardized protocols, scalable manufacturing processes, efficient cargo loading strategies, predictable biodistribution, and harmonized regulatory pathways [116]. The future success of EV-based therapeutics will depend on integrating advances in bioengineering, nanotechnology, manufacturing, regulatory science, and clinical research to address the remaining translational challenges [117]. Continued advances in EV engineering, scalable manufacturing, and clinical development are expected to establish EVs as integral components of precision medicine, targeted drug delivery, and regenerative therapeutics [118].

## Figures and Tables

**Table 1:** Comparison of the major subtypes of extracellular vesicles (EVs): exosomes, microvesicles (MVs), and apoptotic bodies (ApoBDs).

Feature	Exosomes	Microvesicles	Apoptotic Bodies
Size	30–150 nm (smallest)	100–1000 nm	500–5000 nm (largest)
Origin	Endosomal pathway (MVBs)	Plasma membrane budding	Apoptotic cell fragmentation
Main Cargo	Proteins, lipids, RNA, DNA	Proteins, lipids, RNA, DNA	Organelles, DNA fragments, proteins
Primary Function	Cell communication	Cell signaling & inflammation	Clearance of dying cells
Key Surface Markers	CD9, CD63, CD81	Integrins, selectins	Phosphatidylserine, histones
Therapeutic Potential	High	Moderate	Low

**Table 2:** An overview of plant-derived extracellular vesicles (PD-EVs) investigated for the treatment of cancer.

EV Source/Modification	Cancer	Experimental Model	Key Mechanism(s)	Major Therapeutic Outcomes	Reference
Bitter melon-derived EVs (BMEVs); combined with 5-fluorouracil (5-FU)	Oral squamous cell carcinoma (OSCC)	In vitro: OSCC cell lines; In vivo: OSCC tumor model	Induced S-phase arrest and ROS/JUN-mediated apoptosis; downregulated NLRP3; reduced 5-FU resistance; synergized with 5-FU	Suppressed OSCC growth, enhanced 5-FU efficacy, reduced chemoresistance, and showed anti-inflammatory and antitumor activity.	[78]
Ginseng-derived nanoparticles (GDNPs) isolated from Panax ginseng C.A. Mey.	Melanoma (B16F10)	In vitro: M2-polarized macrophages and melanoma cells; In vivo: B16F10 melanoma-bearing mice	Reprogrammed M2 to M1 macrophages via TLR4/MyD88, increased ROS, and enhanced macrophage-mediated melanoma apoptosis.	Suppressed melanoma growth, increased M1 macrophage infiltration, enhanced antitumor immunity, and showed immunotherapeutic potential.	[79]
Ginger-derived exosome-like nanoparticles (GELNs) isolated from ginger rhizomes	Triple-negative breast cancer (TNBC)	In vitro: MDA-MB-231 TNBC cells; normal cell lines	Induced ROS-mediated apoptosis, mitochondrial membrane depolarization, phosphatidylserine externalization, caspase activation, and cell cycle blockade; suppressed cell translocation and colony formation	Selectively reduced TNBC cell viability without affecting normal cells, promoted apoptosis, suppressed cell proliferation, migration, and clonogenic growth	[80]
Tea flower-derived exosome-like nanovehicles (TFENs)	Breast cancer and lung metastasis of breast cancer	In vitro: Breast cancer cell lines; In vivo: Breast tumor-bearing and lung metastasis mouse models	ROS amplification-induced apoptosis; mitochondrial damage; cell cycle inhibition; attenuation of cancer cell proliferation, migration, and invasion; modulation of gut microbiota	Selectively suppressed breast tumor growth, inhibited lung metastasis, accumulated at tumor and metastatic sites following intravenous and oral administration, and exhibited potent anticancer and antimetastatic effects	[81]
Basil-derived EVs (BasEVs) isolated from apoplastic washing fluid (AWF) of *Ocimum basilicum* leaves	Pancreatic cancer	In vitro: MIA PaCa-2 pancreatic cancer cells	Cellular uptake of BasEVs; induction of apoptosis through upregulation of the pro-apoptotic marker Bax at both gene and protein levels; inhibition of clonogenic growth	Significantly reduced pancreatic cancer cell viability and colony formation, promoted apoptosis, and demonstrated potential as a natural anticancer therapeutic and drug delivery platform	[82]
Engineered exosome-like nanovesicles derived from *Asparagus cochinchinensis*	Hepatocellular carcinoma (HCC)	In vitro: HepG2 hepatocellular carcinoma cells; In vivo: HCC tumor-bearing mice	Induced apoptosis and inhibited cancer cell proliferation; enhanced cellular uptake and antitumor activity with low systemic toxicity	Significantly suppressed HCC cell growth and tumor progression while exhibiting a favorable safety profile and reduced toxicity toward normal tissues	[83]

**Table 3:** Representative patents on extracellular vesicle (EV)-based cancer therapeutics incorporating diverse active agents, including publication information and key invention features.

Publication Number/Reference Number	Date of Publication	Patent Jurisdiction	Title	Summary of Invention
116113690 ([CN116113690A])	12 May 2023	China	Composition for promoting production of extracellular vesicles, containing peptide derived from Noxa protein, and method for producing extracellular vesicles using same	A Noxa protein-derived peptide enhances efficient, high-yield EV production and enables therapeutic cargo delivery, including proteins, plasmid DNA, and membrane-impermeable drugs.
WO/2024/225847 ([WO2024225847A1])	31 October 2024	WIPO (PCT)	Method for isolating exosomes or extracellular vesicles derived from breast cancer	Antibody-based methods selectively isolate breast cancer-derived EVs using cancer-specific surface markers, enabling microRNA extraction for diagnostic and research applications.
119060911 ([CN119060911A)]	3 December 2024	China	EVs of lactobacillus salivarius LZZAY01 and application of extracellular vesicles LsEVs in colon cancer	EVs derived from Lactobacillus salivarius LZZAY01 exhibit anti-colon cancer activity while promoting normal intestinal epithelial cell growth, supporting their potential for cancer prevention and therapy.
1020220156460 ([KR1020220156460A])	25 November 2022	South Korea	Immune cells with enhanced secretion of extracellular vesicles, and cancer immunotherapy using same	The invention provides immune cells, including CAR-expressing cells, engineered to enhance EV secretion through MAL and/or CD9 expression, thereby improving the efficacy of cancer immunotherapy.
112870340 ([CN112870340A])	1 June 2021	China	Tumor vaccine based on breast cancer extracellular vesicles and preparation method thereof	A breast cancer EV-based vaccine incorporating Toll-like receptor stimulants and mannose-modified lipids enhances antitumor immunity through immune cell activation, inhibiting tumor growth and supporting cancer immunotherapy.
113528531 ([CN113528531A])	22 October 2021	China	Nucleic acid aptamers for detecting extracellular vesicles of human lung cancer cell strain A549 and application of nucleic acid aptamers	Nucleic acid aptamers enable the specific detection of A549 lung cancer-derived EVs for targeted diagnosis, EV-based drug delivery, and diagnostic applications.
1020240116412 ([KR1020240116412A])	29 July 2024	South Korea	Composition for preventing or treating cancer containing extracellular vesicles (ev) derived from edible crops	Edible crop-derived EVs exhibit intrinsic anticancer activity and serve as natural drug delivery systems, supporting cancer prevention and therapy.

**Table 4:** Clinical trial status of extracellular vesicles in various types of cancer, including study type/phase, study description, enrolment, and clinical trial identifier.

Clinical Trials	Study Type/Phase	Types of Cancer	Description	Enrolment	Identifier
Extracellular Vesicles and Chemotherapy-Induced Peripheral Neuropathy (CHEMOVES)	Interventional	Different types of cancer and chemotherapy-induced peripheral neuropathy	Blood EVs as predictive biomarkers for chemotherapy-induced peripheral neuropathy in cancer patients receiving neurotoxic chemotherapy, through longitudinal analysis of EV levels, lipids, and microRNAs.	120	NCT07558447
Evaluation of Programmed Death Ligand 1 (PDL1) Response to Treatment in EVs, Patient-derived Organoid (PDOs)s and Immune-marker Positron Emission Tomography Scanning in Non-small Cell Lung Cancer	Interventional/Phase 1 and Phase 2	Non-small cell lung cancer	An exploratory pilot study assessed the utility of EVs, patient-derived organoids (PDOs), and PD-L1 PET imaging as biomarkers for predicting therapeutic response to pembrolizumab and dostarlimab in recurrent non-small cell lung cancer (NSCLC).	40	NCT06405230
Olmutinib Trial in T790M (+) NSCLC Patients Detected by Liquid Biopsy Using BALF Extracellular Vesicular DNA	Interventional/Phase 2	Non-small cell lung cancer	A single-arm, open-label Phase II study evaluated the anti-tumor efficacy of Olmutinib (Olita^®^) in T790M-positive NSCLC patients identified using EV-derived DNA from bronchoalveolar lavage fluid.	25	NCT03228277
Unraveling the Role of Extracellular Vesicles-driven Senescence in Myeloproliferative Neoplasms	Observational	Myeloproliferative neoplasms	The study investigates EVs and senescence in myeloproliferative neoplasms to identify sex-specific prognostic biomarkers and personalized therapeutic targets.	110	NCT06798805
Clinical Study of Glycosylated Extracellular Vesicles for Early Diagnosis of Breast Cancer	Observational	Breast cancer	Prospective, single-center cohort study evaluates glycosylated extracellular vesicles (EVs) and their contents as biomarkers for the early detection of breast cancer.	420	NCT05417048
Neoadjuvant Lazertinib Therapy in EGFR-Mutation Positive Lung Adenocarcinoma Detected by BALF Liquid Biopsy	Phase 2	Non-small cell lung cancer	The investigation evaluates neoadjuvant lazertinib in resectable EGFR-mutated NSCLC and explores EV-based bronchoalveolar lavage fluid (BALF) liquid biopsy for non-invasive detection of EGFR mutations.	40	NCT05469022

## Data Availability

No new data were created or analyzed in this study.

## Abbreviations

The following abbreviations are used in this manuscript:

AD	Alzheimers disease
ADSCs-EXOs	Adipose-derived stem cell exosomes
ApoBDs	Apoptotic bodies
APAF-1	Apoptotic peptidase activator factor-1
BBB	Blood–brain barrier
CAA	Cancer-associated adipocytes
CAFs	Cancer-associated fibroblasts
Casp-3	Caspase-3
CCL2	C-C motif chemokine ligand 2
circ-RNA	Circulating-RNA
COLGALT2	Procollagen galactosyltransferase 2
CREB	cAMP response- element binding protein
CRC	Colorectal cancer
EGF	Epidermal growth factor
EGRF1	Epidermal growth factor receptor 1
ESCRT	Endosomal sorting complex required
EVs	Extracellular vesicles
FADD	Fas- associated death domain protein
FAO	Fatty acid oxidation
FGF-1	Fibroblast growth factor-1
FIS-1	Mitochondrial fission protein-1
hADSC	human Adipose-derived stem cell
HIF1-α	Hypoxia-inducible factor 1-α
HISLA	HIF1A stabilizing long noncoding RNA
hMSC	human Mesenchymal stem cells
hTERT	human Telomerase reverse transcriptase
IL-6	Interleukin-6
IL-8	Interleukin -8
ILV	Intraluminal vesicle
ISO	International organization for standardization
JAK	Janus kinase
miR	Micro RNA
MMP-3	Metalloproteinase -3
MMP-9	Metalloproteinase-9
MS	Multiple sclerosis
MSCs	Mesenchymal stem cells
MVs	Multivesicles
MVBs	Multivesicular bodies
NK	Natural killer
OPA-1	Mitochondral dynamin-like GTPase
PD	Parkinsons disease
PD-EVs	Plant-derived extracellular vesicles
PD-L1	Programmed death ligand-1
PSCs	Pancreatic steallate cells
RID-1	GTPase-binding protein rid-1
ROCK	Rho-associated protein kinase
ROS	Reactive oxygen species
SC-EVs	Schwann cell-derived extracellular vesicles
SEVs	Small extracellular vesicles
SOX-9	SRY- box transcription factor-9
STAT-3	Signal transducer and activator of transcription-3
TAMS	Tumor-associated macrophages
TAZ	Taffazin
TEMs	Tetraspanin-enriched microdomains
TDEVs	Tumor derived EVs
TGF-β	Transforming growth factor-β
USP-7	Ubiquitin-specific processing protease-7
VEGF-D	Vascular endothelial growth factor-D
VHL	Von- hippel lindautumor suppressor
YAP	Yes- associated protein 1
